# Supplementary material for: The Biochemical Activities of the Saccharomyces cerevisiae Pif1 Helicase Are Regulated by Its N-Terminal Domain
Source: Genes (Basel). 2019 May 28;10(6):411. doi: 10.3390/genes10060411 (PMC6628001; doi:10.3390/genes10060411)
Supplement: Supplementary file 1 [file genes-10-00411-s001.pdf]

Supplementary Materials

The N-terminal domain of *Saccharomyces cerevisiae* Pif1 is necessary to regulate telomerase activity

David G. Nickens[†], Christopher W. Sausen[†], and Matthew L. Bochman*

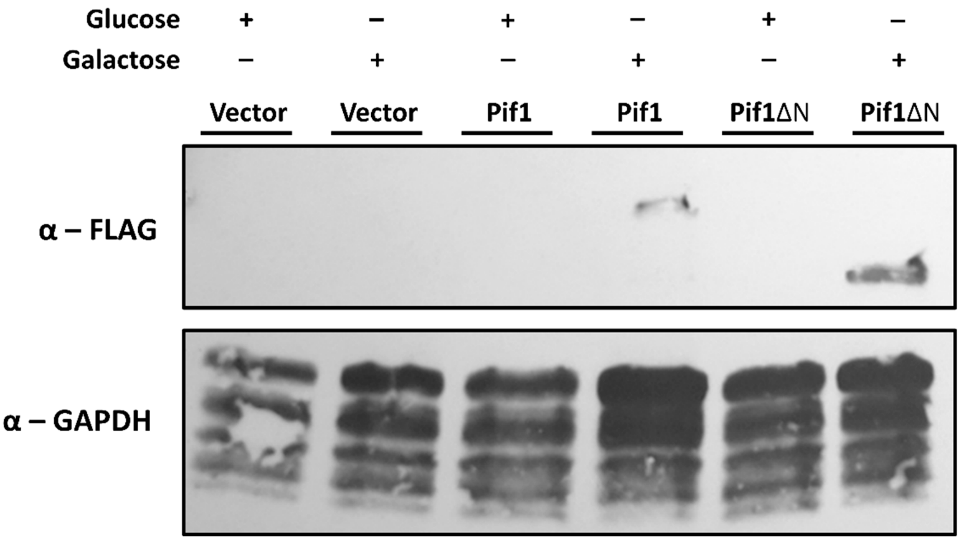

Supplemental Figure S1. Pif1 and Pif1ΔN are overexpressed to similar levels in vivo**.** Wild-type cells were transformed with empty pESC-URA vector (vector) or the same plasmid encoding FLAG-tagged Pif1 or Pif1ΔN under the control of the galactose-inducible *GAL1/10* promoter. Cells were then either grown in media containing glucose (repressive) or galactose (induction), lysed, and blotted with an anti-FLAG antibody. The levels of GAPDH were used as a loading control.

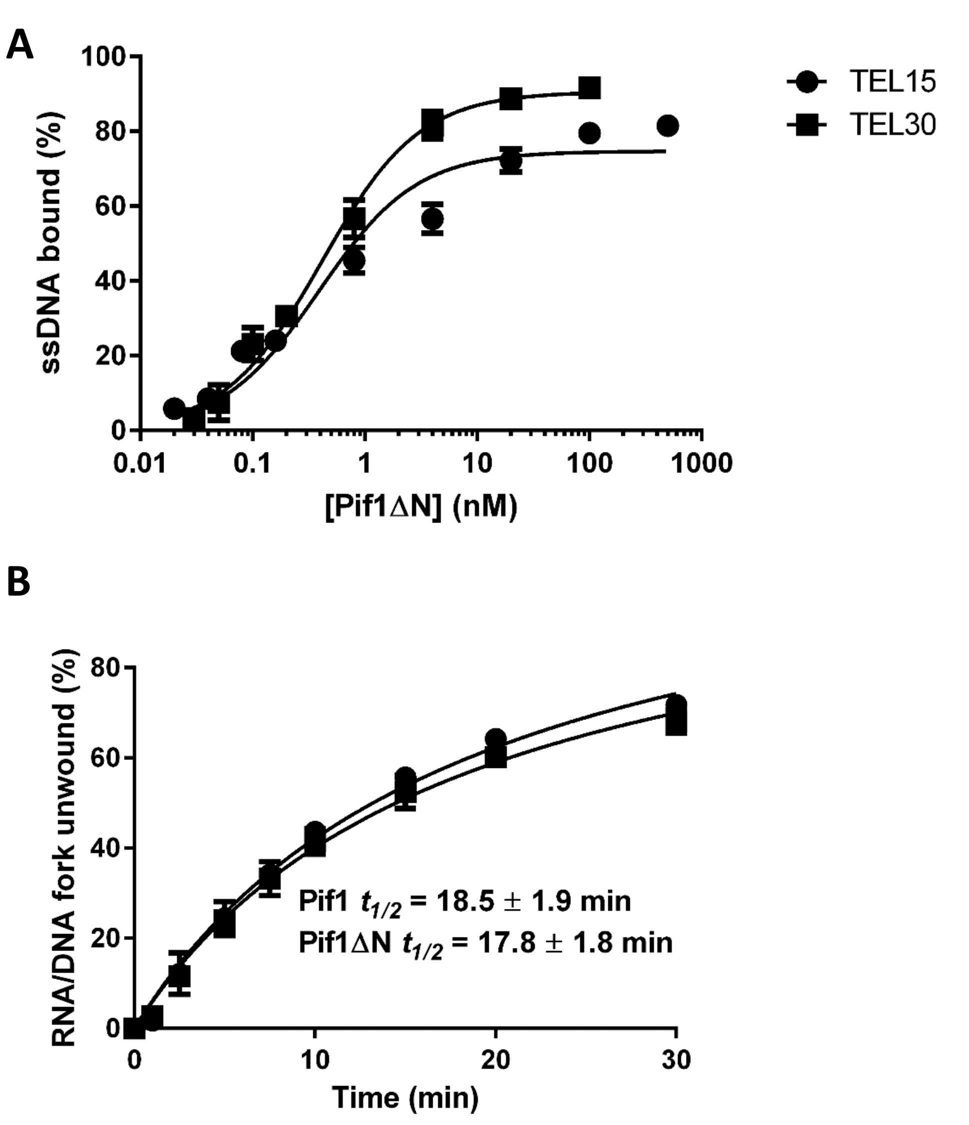

Supplemental Figure S2. Pif1ΔN binds ssDNA tightly and unwinds DNA with similar kinetics to full-length Pif1. (**A**) Pif1ΔN binds the Tel15 and Tel30 ssDNA substrates with similar affinity. The plotted values represent the results of EMSAs using radiolabeled substrates and the indicated concentrations of recombinant Pif1ΔN protein. (**B**) Pif1 and Pif1ΔN unwind forked DNA with similar kinetics. The plotted values represent DNA unwinding time course assays performed with 0.4 nM of a radiolabeled DNA-DNA fork substrate and 2 nM Pif1 or Pif1ΔN. In all cases, the data represent the averages of ≥3 independent experiments, and the error bars are the standard deviation.
